# Eight Bs.

**Published:** 1862-05

**Authors:** 


**EIGHT Bs.**—Who is he? Of any ten men following each other on Broadway, there will be perhaps only one who has enough about him to induce you to either ask his name or his calling; in fact, only one whom you would care to look at a second time. But there comes a quick-stepped, sunny-faced, compact-built, intent-looking man, of forty-five or so, who began life a poor boy; worked so hard that the flesh was worn from his bones; the light of his countenance was gone; and his youthful step became that of feebleness and age; but the fire of life was still in his eye; and an unconquerable will pervaded his whole moral nature. He ate too fast, worked too hard, slept too little, and life's lamp flickered; he tottered on the very verge of an early grave. Then only did he wake up to a rational life; a life of temperate, industrious, and plain, regular habits. Going into his establishment the other day, where, in a six-storied building, a noiseless engine drives thirty power-presses, giving honorable and remunerative employment to three hundred willing and cheerful workers, there was posted on the walls:

"READ and HEED.

The great object of Labor is Profit: to attain that, therefore,

Be Punctual,
Be Industrious,
Be Careful,
Be Quiet,
Be Gentle in Speech,
Be Clean,
Be Obedient,
Be Just."

Whoever then of our young readers wishes to grow up moral, industrious, and successful, can accomplish all these, by putting into habitual and persistent practice the

Eight Bs above. But they are enforced in language so pithy, expressive, and concise, and so quaintly are the moral sentiments appealed to, that there is reason to believe that a good preacher was spoiled when John A. Gray became a printer. And not willing that a composition so suggestive and eminently practical should perish in a hand-bill, we have in another page made our 69th Health Tract of it, by adding some Cs to the Bs.

